# HER2/neu (ERBB2) expression and gene amplification correlates with better survival in esophageal adenocarcinoma

**DOI:** 10.1186/s12885-018-5242-4

**Published:** 2019-01-08

**Authors:** Patrick Sven Plum, Florian Gebauer, Max Krämer, Hakan Alakus, Felix Berlth, Seung-Hun Chon, Lars Schiffmann, Thomas Zander, Reinhard Büttner, Arnulf Heinrich Hölscher, Christiane Josephine Bruns, Alexander Quaas, Heike Loeser

**Affiliations:** 1 Department of General, Visceral and Cancer Surgery, University Hospital Cologne, Cologne, Germany; 2 Institute of Pathology, University Hospital Cologne, Kerpener Str. 62, 50937 Cologne, Germany; 3 Department of Internal Medicine I, University Hospital Cologne, Cologne, Germany; 4 Center for Esophageal and Gastric Surgery, AGAPLESION Markus Krankenhaus, Frankfurt, Germany; 5 Gastrointestinal Cancer Group Cologne (GCGC), University Hospital of Cologne, Kerpener Str. 62, 50937 Cologne, Germany

**Keywords:** HER2/neu (ERBB2), Esophageal adenocarcinoma (EAC), Prognosis, Immunohistochemistry, Tissue microarray (TMA), Fluorescence-in-situ-hybridization (FISH)

## Abstract

**Background:**

HER2 (ERBB2 or HER2/neu) is a tyrosine-kinase increasing cell proliferation. Overexpression/amplification of HER2 is correlated with worse prognosis in solid malignancies. Consequently, HER2 targeting is established in breast and upper gastrointestinal tract cancer. There are conflicting data concerning the impact of HER2 overexpression on esophageal adenocarcinoma (EAC), as most studies do not differ between cancers of the esophagus/gastroesophageal junction and the stomach. The aim of this study was to analyze the expression/amplification of HER2 in EAC in correlation to clinicopathological data to verify its prognostic impact.

**Methods:**

We analyzed 428 EAC patients that underwent transthoracic thoraco-abdominal esophagectomy between 1997 and 2014. We performed HER2 immunohistochemistry (IHC) according to the guidelines and fluorescence-in-situ-hybridization (FISH) for IHC score2+, using tissue micro arrays (TMA) with up to eight biopsies from the surface and infiltration area of a single tumor for evaluating HER2-heterogeneity and single-spot TMA. The HER2-status was correlated with clinicopathological data.

**Results:**

HER2-positivity was found in up to 14.9% in our cohort (IHC score 3+ or IHC score 2+ with gene amplification) and demonstrated a significantly better overall survival (OS) in correlation to HER2-negative tumors (median OS 70.1 vs. 24.6 months, *p* = 0.006). HER2-overexpression was more frequently seen in lower tumor stages (pT1/pT2, *p* = 0.038), in the absence of lymphatic metastases (pN0/pN+, *p* = 0.020), and was significantly associated with better histological grading (G1/G2) (*p* = 0.041).

**Conclusion:**

We demonstrated a positive prognostic impact of HER2 overexpression in a large cohort of EAC, contrary to other solid malignancies including gastric cancer and breast cancer, but consistent to the results of a large study on EAC from 2012.

## Background

Esophageal adenocarcinoma (EAC) is a fatal disease with high mortality and increasing incidences in the Western world [1–3]. Although therapeutic procedures and treatment concepts have evolved, resulting in a trimodality therapy including neoadjuvant chemoradiation followed by radical oncological surgery, prognosis still remains limited [4–6]. Significant efforts have been made in this field, resulting in the description of a large variety of different putative markers, but so far, only one of them has made its way into the guidelines for targeted therapy for patients with advanced gastroesophageal adenocarcinoma, namely the human epidermal growth factor receptor 2 (HER2) [7].

The HER2 (also known as ERBB2 or HER2/neu) is a member of the epidermal growth factor receptor (EGFR) family and encodes a 185-kDa transmembrane tyrosine kinase receptor [8, 9]. Physiologically, HER2 is expressed in several tissues such as the nervous system, epithelial cells, or the mammary gland, where it promotes cell proliferation, controls differentiation, or suppresses apoptosis [9–11]. In case of uncontrolled activation of its associated pathway, this might result in excessive cell growth, angiogenesis, and tumorigenesis [11, 12]. Until today, HER2-overexpression/gene amplification has been detected in multiple solid tumor entities including breast cancer, lung cancer, glioblastoma, head and neck cancer, pancreatic cancer, colorectal cancer, gastric cancer, or EAC [12]. Trastuzumab is a humanized monoclonal antibody selectively targeting against HER2 at its extracellular domain of the receptor, resulting in an antibody-mediated cellular cytotoxicity [13]. In patients with HER2-positive advanced gastric or esophagogastric junction adenocarcinoma, the use of trastuzumab in combination with chemotherapy is a standard therapy concept and has a positive prognostic effect compared to chemotherapeutic treatment alone [14]. However, current data considering the prognostic role of HER2 in EAC are still controversial [15–19]. According to the current literature, the rate of HER2 positivity in EAC varies, ranging from 15 to 29% [19–24].

In this context, the aim of the present study is to analyze the expression/gene amplification as well as the distribution of HER2 in our large EAC cohort in correlation to the corresponding clinicopathological data, with the aim to verify the incidence and prognostic impact in this specific tumor subgroup.

## Methods

### Patients and tumor samples

To assess the distribution and heterogeneity of HER2, we created a multi-spot tissue array (TMA) with 165 tumor cases, according to the suggestions of the international immunooncology working group for assessing tumor infiltrating lymphocytes (TILs) in solid tumors. Four tissue cores from each tumor were punched out (diameter 1.2 mm) from the tumor surface/−center and from the deep-infiltration margin, respectively, and transferred into a TMA recipient block. For more detailed information, see Simon et al. and Helbig et al. [25, 26]. We defined the infiltration margin as the tumor areal showing the widest spatial distance from the mucosa-surface. In a second step, we used a TMA with single tumor punches of a further collective of 428 tumor cases for validating our HER2 analyses from the multi-spot TMA. For this, 4-μm sections of the resulting TMA blocks were transferred to an adhesive-coated slide system (Instrumedics Inc., Hackensack, NJ) for standard HE-staining. This retrospective study was performed according to the criteria of the ethics committee of the University Hospital of Cologne.

### Immunohistochemistry

**Fig. 1:**
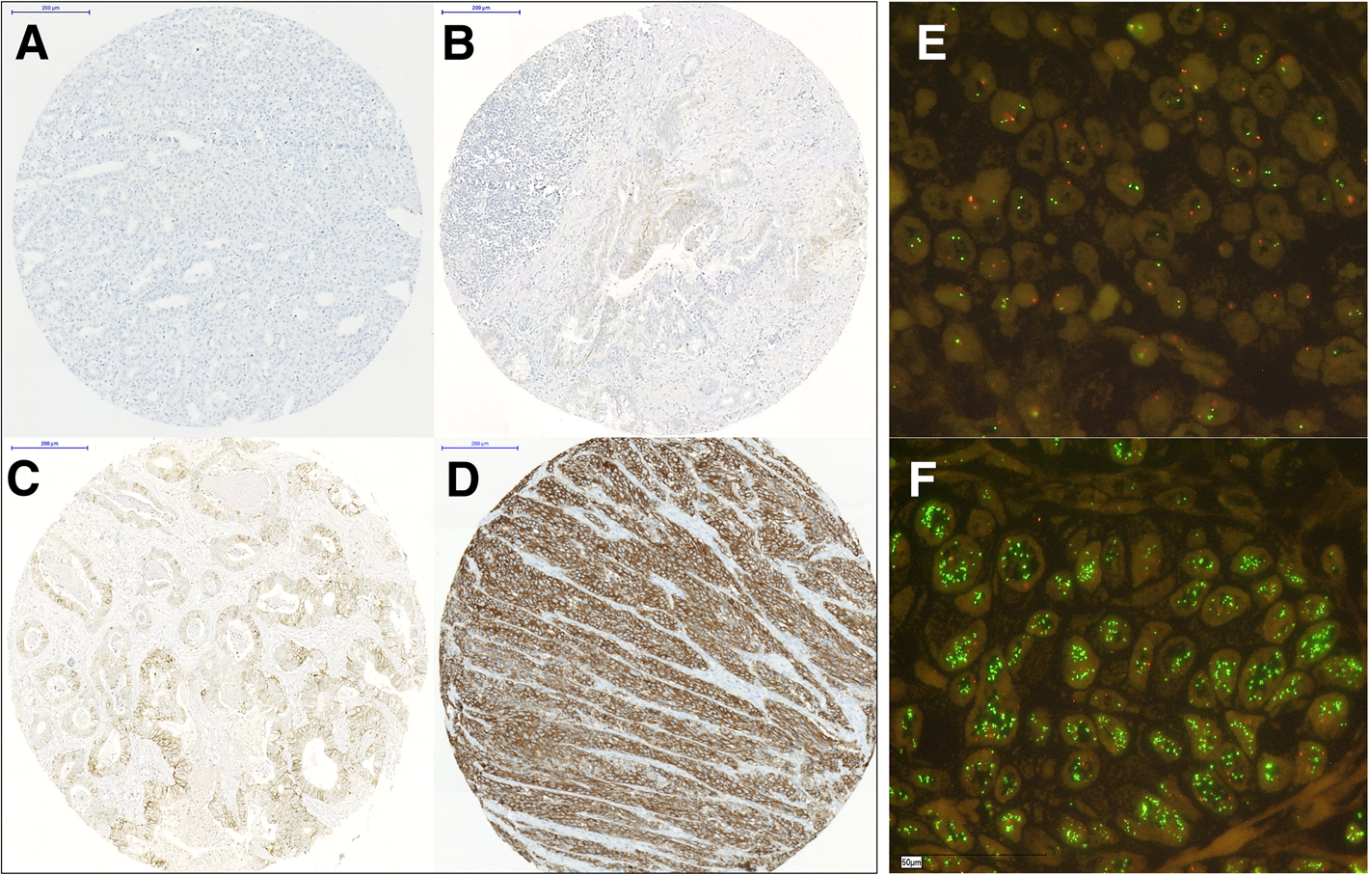
Representative images of histopathological slides to illustrate the immunohistochemistrical scoring system **a**-**d**) as well as FISH-analyses (**e**, **f**): **a**) Negative or staining in < 5 cells (score 0); **b**) very weak staining in cell groups ≥5 (score 1+); **c**) weak to moderate complete/basolateral/lateral staining in cell groups ≥5 (score 2+); **d**) strong complete/basolateral/lateral staining in cell groups ≥5 (score 3+). Representative FISH-specimens **e**) without and **f**) with HER2 amplification

Immunohistochemistry (IHC) was performed on TMA slides. For HER2, the Federal Drug Association (FDA) approved ready-to-use antibody (Pathway anti-Her2/neu rabbit monoclonal primary antibody, clone 4B5, Ventana) on the automated Ventana/Roche slide stainer was used. Membranous expression of HER2 in carcinoma cells was assessed according to the criteria for biopsies [27]: negative or staining in < 5 cells (score 0); very weak staining in cell groups ≥5 (score 1+); weak to moderate complete/basolateral/lateral staining in cell groups ≥5 (score 2+); strong complete/basolateral/lateral staining in cell groups ≥5 (score 3+). Scores 0 and 1+ display negative HER2 status, and score 3+ indicates positive HER2 status. Score 2+ has to be analyzed further by fluorescence or chromogenic in situ hybridization (see Fig. 1). The evaluation of immunohistochemical expression was assessed manually by two pathologists (A.Q. and H.L.). Discrepant results, which occurred only in a small number of samples, were resolved by consensus review.

### Fluorescence in-situ hybridization

Fluorescence in-situ hybridization (FISH) for the evaluation of the HER2 gene amplification status was performed with the Zytolight SPEC ERBB2/CEN 17 Dual Probe Kit (Zytomed, Systems GmbH, Germany) according to the manufacturers’ protocol. Sample processing was performed as previously described [28]. Tumor tissue was scanned for amplification hot spots using a 63x objective (DM5500 fluorescent microscope; Leica). In case the signals were homogeneously distributed, then random areas were used for counting the signals. Twenty tumor cells were evaluated by counting green *HER2* and orange centromere 17 (*CEN17*) signals. The reading strategy followed the recommendations *HER2/CEN17* ratio ≥ 2.0 or *HER2* signals ≥6.0 and negative ratio (< 2.0) [29].

### Statistical analysis

Clinical data were collected prospectively according to a standardized protocol. For statistical analysis, SPSS Statistics for Mac (Version 21, SPSS) was used. Interdependence between staining and clinical data was calculated using the chi-squared and Fisher’s exact tests and displayed by cross-tables. Survival curves were plotted using the Kaplan-Meier method and analyzed using the log-rank test. The heatmap for visualization was generated via Microsoft Excel for Mac; it is considered to visualize the heterogeneity within the tumor and is not a heatmap as commonly used for gene signatures (e.g., next-generation-sequencing (NGS)). Univariate and multivariate analyses were performed for prognostic factors of overall survival, using the Cox regression model. All tests were two-sided; *P* values < 0.05 were considered statistically significant.

## Results

### Patients’ baseline characteristics

A total of 362 patients of 428 on the TMA with EAC that underwent surgical tumor resection were immunohistochemically interpretable on the single-spot and 161 patients on the multi-spot TMA. Reasons for non-informative cases (66 spots; 15.4% on the single-spot TMA, 5 spots 2.4% on the multi-spot TMA) included lack of tissue samples or absence of unequivocal cancer tissue in the TMA spot. Operative procedures were either thoraco-abdominal en-bloc esophagectomy (*n* = 274, 64.0%) with intrathoracic anastomosis or transhiatal esophagectomy with transabdominal or cervical anastomosis (*n* = 154, 36.0%). Clinicopathological data is summarized in Table 1a. Median age at time of operation of the entire patient cohort was 64.9 years (range 33.6–84.5 years, average age 62.4 years, standard deviation +/− 10.7 years). The cohort was split into two groups according to age, above and below the median age, to conduct cross-table analysis revealing possible associations between patient age and clinical, respectively histochemical, data (see Table 1b). In total, 59.8% of the entire patient cohort received any kind of neoadjuvant treatment. Chemoradiation therapy was administered in 68.9% of those patients according to the CROSS protocol or modified CROSS, according to the individual patient’s performance (reduction of either chemo- or radiation dosage). The remaining patients received chemotherapy upfront surgery according to the FLOT or the ECF protocol. On the single-spot TMA, 42 patients (9.8%) were female and 386 (90.2%) were male; a similar distribution was found on the multi-spot TMA (90.3% male, 9.7% female) The median age of the entire patient cohort was 65.2 years (range 33.6–85.6 years) at time of diagnosis. Neoadjuvant treatment (chemo- or radiochemotherapy) was administered to 253 patients (59.1%) before operation on the single-spot TMA and to 23 patients (13.9%) on the multi-spot-TMA.

**Table 1:** Clinicopathological data of the EAC-patients included in the study

				HER2 expression				*P* value
		Total		Negative		Positive		
a) Demographic and pathological results of the cohort								
SEX	female	38	10.5%	34	89.5%	4	10.5%	0.333
	male	324	89.5%	284	87.70%	40	12.3%	
Age group	< 65 yrs	195	53.8%	167	85.5%	28	14.5%	0.062
	> 65 yrs	167	46.1%	151	90.4%	16	9.6%	
Tumor stage	pT1	33	9.1%	26	80.0%	7	20.0%	0.038
	pT2	30	8.3%	28	93.9%	2	6.1%	
	pT3	287	79.2%	251	87.7%	35	12.3%	
	pT4	11	3.0%	11	100.0%	0	0.0%	
Lymph node metastasis	pN0	138	38.0%	113	82.5%	24	17.5%	0.02
	pN+	224	62.0%	204	91.1%	20	8.9%	
Grading	G1/2	152	61.8%	126	82.9%	26	17.1%	0.009
	G3/4	94	38.2%	89	94.7%	5	5.4%	
UICC	I	50	13.8%	39	78.8%	11	21.2%	0.039
	II	53	14.6%	47	89.1%	6	10.9%	
	III	169	46.7%	147	86.9%	22	13.1%	
	IV	89	24.6%	84	94.7%	5	5.3%	
b) Cross-table analysis of the patient cohort								
	Hazard ratio			95% confidence interval				*P* value
				Lower	Upper			
SEX (female vs. male)	1.718			0.977	3.023			0.06
Age group (< 65 vs. > 65 yrs)	1.194			0.914	1.559			0.193
pT (pT1/2 vs. pT3/4)	1.331			0.982	1.804			0.065
pN (pN0 vs. pN+)	0.937			0.77	1.139			0.513
UICC (Stage I/II vs. III/IV)	1.975			1.559	2.503			0
HER2 expression (neg. vs. pos.)	0.628			0.401	0.983			0.042

### HER2 expression

The HER2 immunostaining was localized in the membranes of tumor cells. In total, HER2 positivity was detectable in 12.2% (*n* = 44) of interpretable EAC cases on the single-spot TMA. On the multi-spot TMA, HER2 expression was found in 24 patients (14.9%) on the surface area and in 18 patients (10.9%) at the infiltration margin.

**Fig. 2:**
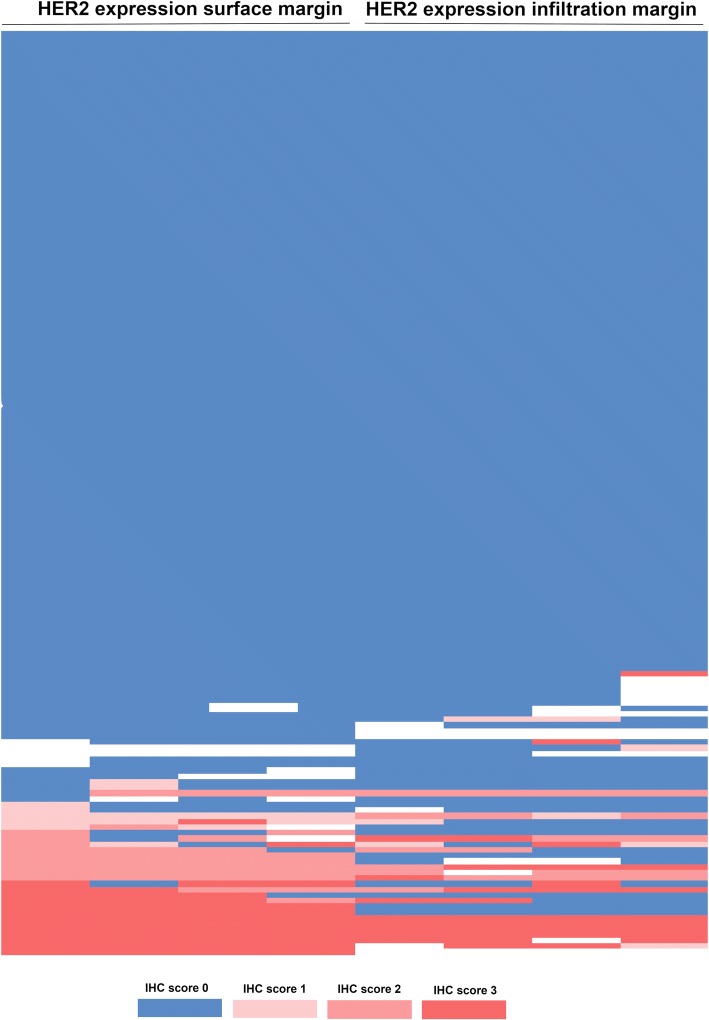
Heatmap showing heterogeneity of HER2 expression between the luminal and the infiltration area of the primary tumor. Blue area represents absence of HER2 expression (IHC score 0); light red immunohistochemistry (IHC) score 2+, FISH confirmation negative; medium red IHC score 2+, FISH positive; dark red IHC score 3 +

The HER2 expression was correlated with lower pT-stages (pT1/2 vs. pT3/4, *p* = 0.038), low-grade stages (G1/2 vs. G3/4 *p* = 0.041), and the absence of lymph node metastasis (pN0 vs. pN+, *p* = 0.020). This reflects a correlation with early UICC stages, which was also significantly correlated (*p* = 0.039). In patients who underwent neoadjuvant treatment, HER2 expression was seen in a higher frequency than in patients without neoadjuvant treatment (21.2% vs. 9.2%, *p* = 0.027). Considering the heterogeneity of the HER2 expression, there was no significant difference between the surface and infiltration margins of the EAC specimens analyzed with a strong correlation between the expression status on the surface and infiltration margin (Fig. 2). A total of 133 patients were negative in both areas on the multi-spot TMA (84.2%), while 16 patients were double-positive (11.4%) (*p* < 0.0001).

### HER2 as a prognostic biomarker

**Fig. 3:**
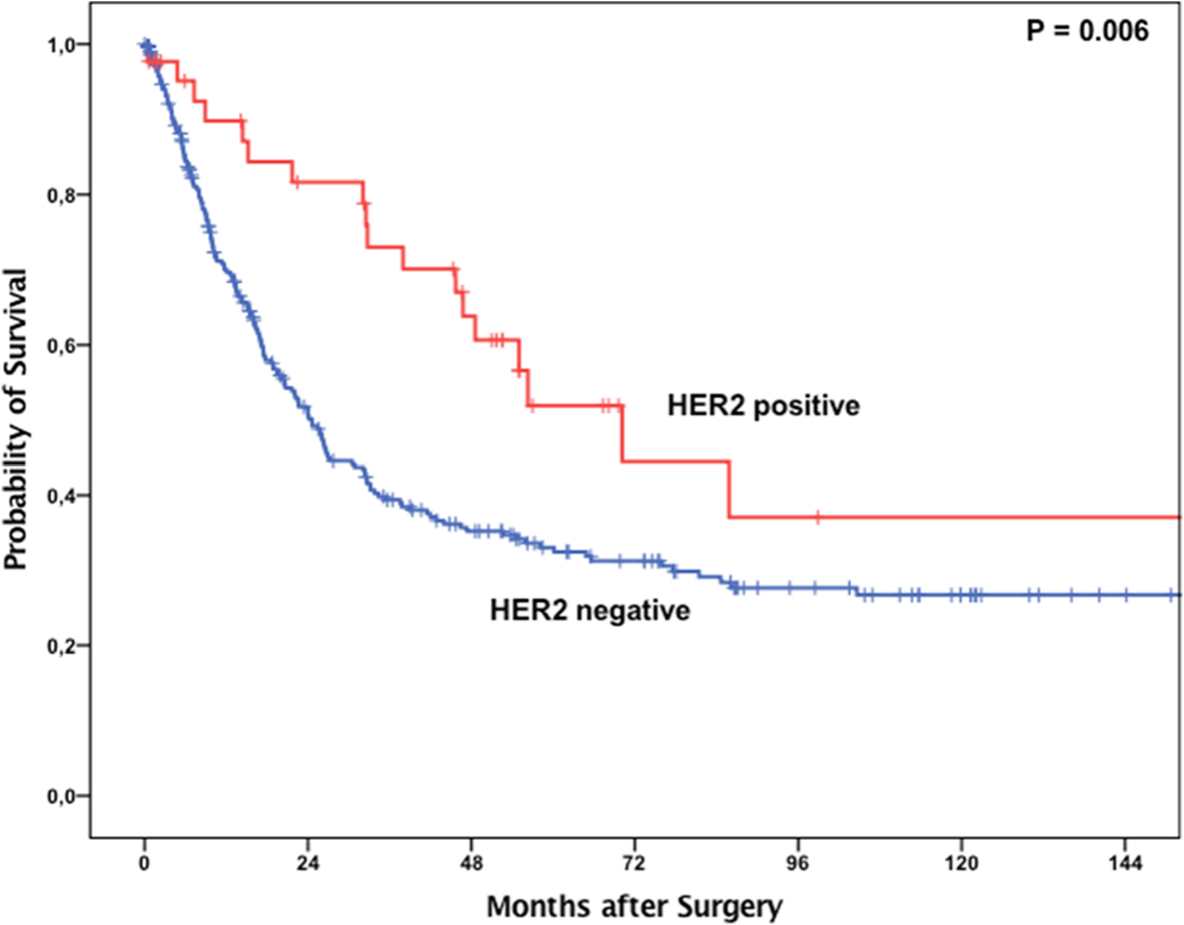
Kaplan-Meier survival analysis (log-rank test) for the entire patient cohort on the single-spot TMA. Significant survival difference between patients with HER2 expression (median OS 70.1 months (95% confidence interval 44.0–95.6 months) compared to patients without HER2 expression (median OS 24.6 months (95% confidence interval 20.7–28.5 months), *p* < 0.006))

Patients with HER2 expression showed a superior overall survival (OS) compared to HER2-negative tumors. The median OS was 70.1 months (95% confidence interval (CI) 44.0–95.6 months) in HER2-positive tumors compared to a median OS of 24.6 months (95%CI 20.7–28.5 months, *p* = 0.006) in HER2-negative cases (Fig. 3). Patients that underwent neoadjuvant therapy showed lower HER2 expression than primarily resected patients (primary surgery 21% vs. neoadjuvant treated 9%). In the subgroup analysis, HER2 prognostic survival difference was only seen in the group of patients who underwent neoadjuvant treatment and not in the primary resected group.

### Multivariate analysis

Multivariate cox-regression analysis revealed, beside the UICC stage, HER2 expression as independent prognostic with a hazard ratio of 0.628 (95% CI 0.401–0.983), displaying an improved OS in cases of HER2 expression (*p* = 0.042).

## Discussion

A large diversity of different putative diagnostic or predictive biomarkers has been considered for EAC in the recent past. At least the status of HER2 is by now considered as a predictive marker for trastuzumab therapy in advanced gastric and gastroesophageal cancer, since a prognostic therapeutic effect has been shown in the ToGA-study [14]. The HER2 physiologically controls cell differentiation or promotes cell growth via growth factor-induced signal transduction in several tissues. Consequently, dysregulated HER2 may cause tumorigenesis by suppressing apoptosis and by other effects [10–12]. A high expression of HER2 can be found in several solid tumor entities such as breast cancer, colorectal cancer, lung cancer, or pancreatic cancer [12]. The results considering HER2 in EAC within the upper gastrointestinal tumors are conflicting at first view, as most of the studies do not clearly distinguish between cancers of the esophagus/gastroesophageal junction and the stomach [24, 30–33]. In gastric cancer, HER2 overexpression is associated with poor survival and worse prognosis [12].

We found HER2 positivity in 14.9% (multi-spot TMA) and 12.2% (single-spot TMA) in our tumor cohort. This corresponds with the data published in literature reporting HER2 amplification/overexpression in 11–29% of all EAC-patients considered per analysis and identified via immunohistochemistry (IHC) and fluorescence-in-situ-hybridization (FISH), as done in the current study [18–24]. Both methods work well together, since a concordance of 93.5% between IHC and FISH has been described previously [34]. The variance of HER2 frequency is in mainly explained by different technical issues and divergent evaluation criteria as well as the low number of cases. We found comparable rates of HER2 overexpression/amplification in a large and well defined cohort of EAC [19]. Referring to the literature, HER2 immunoscoring differs not only between breast carcinoma and upper gastrointestinal adenocarcinoma, but there are different criteria for biopsies and surgical specimens of EAC/gastric adenocarcinoma (Table 2). Besides these conventional histopathological evaluation methods, the results of the current study correlate with the genetic data of Dulak et al., who performed NGS on 149 surgical specimens of patients with primary resected esophageal adenocarcinomas or cancers of the esophagogastric junction [35]. In their sequencing study, they identified HER2 mutation in 3% and HER2 amplification in 19% of the cases [35]. Similar results have been presented in 2017 by the Cancer Genome Atlas Research Network. Within their comprehensive molecular analysis of upper gastrointestinal adenocarcinomas, including 77 EAC tumors from patients who underwent primary resection, the authors demonstrated that HER2 amplifications took place in 19 cases (24.68%), while HER2 mutations occurred in three patients (3.9%). In an additional six cases of EAC (7.79%), multiple alterations of HER2 were detectable [36]. But not only in tissue specimens, but also in circulating tumor DNA, genomic alterations of HER2 were found. Therefore, Kato et al. have analyzed 55 patients with advanced gastroesophageal adenocarcinomas via NGS, considering single nucleotide variants, copy number amplifications, fusions, and indels in selected genes [37]. Most of those patients did not qualify for surgical resection (*n* = 46), while 42 patients (67%) showed alternated circulating tumor DNA. Furthermore, the authors described HER2 alterations in eight cases (14.5%) of their study cohort.

**Table 2:** Differences between HER2 evaluation in tumors of the upper gastrointestinal tract and breast cancer

	Tumors of the upper gastrointestinal tract	Breast cancer
Threshold	≥ 5 positive tumor cells of biopsies; ≥ 10% surgical specimen	≥ 10% positive tumor cells
Expression pattern	(baso)-lateral	circular

In gastric and esophageal adenocarcinoma, HER2 shows a heterogeneous intratumoral distribution pattern; thus, some authors recommend a minimum of five biopsies to predict a precise HER2 status [34, 38, 39]. A recent study of HER2 expression in gastric adenocarcinoma and EAC revealed a heterogeneous expression in 27% of 15 paired biopsy and resection specimen, although only two of them showed a different overall HER2-status [40]. We therefore built our multi-spot TMA with up to eight tumor biopsies from the surface and infiltration area with comparable amounts of cancer cells according to endoscopically obtained biopsies to gain a reliable representation of the tumor heterogeneity, even exceeding the recommendation of five biopsies. In our cohort, we found no relevant heterogeneous expression of HER2, considering the spots of the multi-spot TMA neither within the same localization (infiltrative margin or surface) nor within the entire tumor (Fig. 1).

In the literature, most studies proclaimed a negative impact of HER2 on the patients’ prognosis, as first described in breast carcinoma and which dramatically changed the standard therapy for numerous patients with the implementation of trastuzumab in the clinical routine [41, 42]. Many of the studies on gastrointestinal malignancies did not differ between different adenocarcinoma entities of the upper gastrointestinal tract, thus focusing on the prognostic impact of HER2 in EAC alone reveals ambivalent results [30–33]. Moreover, the therapeutic effects of a HER2 blockade in gastrointestinal tumors were rather disappointing compared to the strong benefits in breast cancer. In 2011, Langer et al. have demonstrated a significant negative prognostic effect of HER2 in 142 EAC for disease-free survival as well as overall survival and an association with poor tumor differentiation [20]. In a meta-analysis of 14 studies with either EAC or esophageal squamous cell carcinoma, the authors found a significantly poorer survival rate of HER2-positive EAC patients in studies with over 100 patients [22]. Consequently, the authors postulated HER2 to be a negative prognostic indicator in this context. A recently published analysis by Kato et al. drew the same conclusion in their NGS study of a total of 55 patients with mostly irresectable gastroesophageal adenocarcinomas [37]. Multivariate analysis in those irresectable patients revealed that detectable HER2 mutations within circulating tumor DNA were significantly associated with a poor overall survival compared to patients with the HER2 wild-type (*p* = 0.003) [37]. However, as mentioned before, maybe due to the small numbers of patients, the authors among Kato included different tumor entities within their cohort: On a closer look, only 11 patients had actual EAC tumors, while the other cases showed either gastric or gastroesophageal cancers [37]. Therefore, the transferability of these results to EAC in general seems to be limited in our opinion. In a published genomic characterization, the Cancer Genome Atlas Research Network identified no prognostic difference (*p* = 0.781) between those patients with HER2 amplification/alteration in upper gastrointestinal adenocarcinomas compared to those without (median overall survival: 31.28 vs. 28.75 months) [36]. Focusing on actual EAC tumors within the TCGA data set of this study, prognoses of 73 patients were available. Still, overall survival of this patient subgroup was not significantly altered in correlation to the HER2 mutation/amplification level (*p* = 0.571).

In contrast, another study, currently the largest one, considered an EAC cohort of 713 patients analyzed by Yoon et al., identifying HER2 positivity to be associated with better disease-specific survival and overall survival [19]. These results are fully in line with the results of our tumor cohort. From our point of view, HER2 expression in EAC indicates a biological favorable tumor behavior (early stage of disease, negative lymph node metastasis) and therefore hints to a certain tumor subgroup, associated with a better prognosis per se. This hypothesis is supported by the results of Yoon et al. [19].

In their work, Yoon et al. have demonstrated, via multivariate analysis, that the overall survival (*p* = 0.0022) as well as the disease-specific survival (*p* = 0.0065) among EAC patients who also had Barrett esophagus were independent of pathologic features such as tumor grade, depth of invasion, nodal status, and tumor location [19]. We obtained similar results within our study cohort: Multivariate cox-regression analysis revealed HER2 overexpression/amplification as an independent prognostic factor considering the overall survival.

Interestingly, the cohort of Yoon et al. included only patients who underwent primary surgery, while our study also considered patients with neoadjuvant therapy. This is concordant to the literature, where the differences of the HER2 status in pre-treatment biopsies compared to post-treatment surgical specimens are quantified with 5.9 and 6%, respectively [43, 44]. However, in a subgroup analysis, those patients who underwent neoadjuvant therapy showed lower HER2 positivity compared to the patients with primary resection, and the HER2 prognostic survival difference was only seen in the group of patients with neoadjuvant treatment. Thus, the data are also more consistent, since HER2 is expressed in early carcinomas, nodal-negative, and G1/2 patients, and those are treated neoadjuvantly less frequently.

Although HER2 is a positive prognostic marker in EAC in our cohort, these tumors are still able to metastasize, and consequently, a selectively targeted therapy with trastuzumab should be initiated since it significantly improves the patients’ prognosis compared to conventional chemotherapy alone [14]. However, hypothetically, the positive prognostic impact does not only derive from the pharmacological blockade of HER2, but also by those effects of the HER2-positive tumor subgroup itself.

With regard to the aspect of survival differences between patients with and without neoadjuvant therapy, the present study design naturally shows certain limitations. Patients with complete histopathological regression cannot be considered on the TMA due to missing tumor tissue and are therefore not included in the survival analysis. In addition, patients in advanced tumor stages are treated neoadjuvantly; those patients have a worse overall survival in advance than patients in early tumor stages who were not treated neoadjuvantly.

The major strengths of the current analysis are its large cohort size of patients with well-characterized EAC, including long-term follow-up data. By using TMA specimens for analysis due to the HER2 homogeneity detected in our cohort, this study also models the analysis of biopsy specimens, in which similar amounts of patient-derived materials are available for further diagnostics.

## Conclusions

In conclusion, this study indicates the positive biological effects of HER2 positivity in EAC, being associated with better prognosis, earlier tumor stages, and a lower rate of lymphatic metastasis, representing a hitherto insufficiently characterized subtype of EAC.

## Abbreviations

CEN17: Centromere 17
EAC: Esophageal adenocarcinoma
FDA: Federal Drug Association
FISH: Fluorescence-in-situ-hybridization
GCGC: Gastrointestinal Cancer Group Cologne
HER2: Human epidermal growth factor receptor 2
IHC: Immunohistochemistry
NGS: Next-generation sequencing
OS: Overall survival
TILs: Tumor infiltration lymphocytes
TMA: Tissue micro arrays

## References

[1] Arnold M, Laversanne M, Brown LM, Devesa SS, Bray F. Predicting the future burden of esophageal cancer by histological subtype: international trends in incidence up to 2030. Am J Gastroenterol. 2017;112(8):1247–1255. https://www.ncbi.nlm.nih.gov/pubmed/?term=28585555. 10.1038/ajg.2017.155 28585555

[2] Enzinger PC, Mayer RJ. Esophageal cancer. N Engl J Med. 2003;349(23):2241–2252. https://www.ncbi.nlm.nih.gov/pubmed/?term=14657432. 10.1056/NEJMra035010 14657432

[3] Lagergren J, Smyth E, Cunningham D, Lagergren P. Oesophageal cancer. 390, Lancet. 2017. p. 2383–2396. https://www.ncbi.nlm.nih.gov/pubmed/?term=28648400. 10.1016/S0140-6736(17)31462-9 28648400

[4] Dai T, Shah MA. Chemoradiation in oesophageal cancer. Best Pract Res Clin Gastroenterol. 2015;29(1):193–209. https://www.ncbi.nlm.nih.gov/pubmed/?term=25743466. 10.1016/j.bpg.2014.11.006 25743466

[5] van Hagen P, Hulshof MCCM, van Lanschot JJB, Steyerberg EW, Henegouwen MIVB, Wijnhoven BPL (2012). Preoperative chemoradiotherapy for esophageal or junctional cancer. N Engl J Med.

[6] Xi M, Hallemeier CL, Merrell KW, Liao Z, Blum Murphy MA, Ho L, et al. Recurrence Risk stratification after preoperative chemoradiation of esophageal adenocarcinoma. Ann Surg. 2018 Aug;268(2):289-295. https://www.ncbi.nlm.nih.gov/pubmed/?term=28628563. 10.1097/SLA.0000000000002352 28628563

[7] Bartley AN, Washington MK, Ventura CB, Ismaila N, Colasacco C, Benson AB, et al. HER2 testing and clinical decision making in gastroesophageal adenocarcinoma: guideline from the College of American Pathologists, American Society for Clinical Pathology, and American Society of Clinical Oncology. Arch Pathol Lab Med. 2016;140(12):1345–1363. https://www.ncbi.nlm.nih.gov/pubmed/?term=27841667. 10.5858/arpa.2016-0331-CP 27841667

[8] Akiyama T, Sudo C, Ogawara H, Toyoshima K, Yamamoto T. The product of the human c-erbB-2 gene: a 185-kilodalton glycoprotein with tyrosine kinase activity. Science. 1986;232(4758):1644–1646. https://www.ncbi.nlm.nih.gov/pubmed/?term=3012781. 10.1126/science.3012781 3012781

[9] Casalini P, Iorio M V., Galmozzi E, Ménard S. Role of HER receptors family in development and differentiation. J Cell Physiol. 2004 Sep;200(3):343-50. https://www.ncbi.nlm.nih.gov/pubmed/?term=15254961. 10.1002/jcp.20007 15254961

[10] Rubin I, Yarden Y. The basic biology of HER2. Ann Oncol. 2001;12 Suppl 1:S3-8. https://www.ncbi.nlm.nih.gov/pubmed/?term=11521719. 10.1093/annonc/12.suppl_1.s3 11521719

[11] Ménard S, Pupa SM, Campiglio M, Tagliabue E. Biologic and therapeutic role of HER2 in cancer. Oncogene. 2003;22(42):6570–6578. https://www.ncbi.nlm.nih.gov/pubmed/?term=14528282. 10.1038/sj.onc.1206779 14528282

[12] Roskoski R. The ErbB/HER family of protein-tyrosine kinases and cancer. Pharmacol Res. 2014 Jan;79:34-74. https://www.ncbi.nlm.nih.gov/pubmed/24269963. 10.1016/j.phrs.2013.11.002 24269963

[13] Hudis CA. Trastuzumab--mechanism of action and use in clinical practice. N Engl J Med. 2007;357(1):39–51. https://www.ncbi.nlm.nih.gov/pubmed/?term=17611206. 10.1056/NEJMra043186 17611206

[14] Bang YJ, Van Cutsem E, Feyereislova A, Chung HC, Shen L, Sawaki A, et al. Trastuzumab in combination with chemotherapy versus chemotherapy alone for treatment of HER2-positive advanced gastric or gastro-oesophageal junction cancer (ToGA): A phase 3, open-label, randomised controlled trial. Lancet. 2010;376(9742):687–697. https://www.ncbi.nlm.nih.gov/pubmed/?term=20728210. 10.1016/S0140-6736(10)61121-X 20728210

[15] Shitara K, Ikeda J, Kondo C, Takahari D, Ura T, Muro K, et al. Reporting patient characteristics and stratification factors in randomized trials of systemic chemotherapy for advanced gastric cancer. Gastric Cancer. 2012;15(2):137–143. https://www.ncbi.nlm.nih.gov/pubmed/?term=21842172. 10.1007/s10120-011-0083-8 21842172

[16] Janjigian YY, Werner D, Pauligk C, Steinmetz K, Kelsen DP, Jäger E, et al. Prognosis of metastatic gastric and gastroesophageal junction cancer by HER2 status: A European and USA International collaborative analysis. Ann Oncol. 2012;23(10):2656–2662. https://www.ncbi.nlm.nih.gov/pubmed/?term=22689179. 10.1093/annonc/mds104 22689179

[17] Okines AFC, Thompson LC, Cunningham D, Wotherspoon A, Reis-filho JS, Langley RE, et al. Effect of HER2 on prognosis and benefit from peri-operative chemotherapy in early oesophago-gastric adenocarcinoma in the MAGIC trial. Ann Oncol. 2013;24(5):1253–1261. https://www.ncbi.nlm.nih.gov/pubmed/?term=23233651. 10.1093/annonc/mds622 23233651

[18] Reichelt U, Duesedau P, Tsourlakis MC, Quaas A, Link BC, Schurr PG, et al. Frequent homogeneous HER-2 amplification in primary and metastatic adenocarcinoma of the esophagus. Mod Pathol. 2007;20(1):120–129.https://www.ncbi.nlm.nih.gov/pubmed/?term=17143264. 10.1038/modpathol.3800712 17143264

[19] Yoon HH, Shi Q, Sukov WR, Wiktor AE, Khan M, Sattler CA (2012). Association of HER2/ErbB2 expression and gene amplification with pathologic features and prognosis in esophageal adenocarcinomas. Clin Cancer Res.

[20] Langer R, Rauser S, Feith M, Nährig JM, Feuchtinger A, Friess H, et al. Assessment of ErbB2 (Her2) in oesophageal adenocarcinomas: Summary of a revised immunohistochemical evaluation system, bright field double in situ hybridisation and fluorescence in situ hybridisation. Mod Pathol. 2011;24(7):908–916. https://www.ncbi.nlm.nih.gov/pubmed/?term=21516080. 10.1038/modpathol.2011.52 21516080

[21] Koopman T, Smits MM, Louwen M, Hage M, Boot H, Imholz ALT. HER2 positivity in gastric and esophageal adenocarcinoma: clinicopathological analysis and comparison. J Cancer Res Clin Oncol. 2015;141(8):1343–1351. https://www.ncbi.nlm.nih.gov/pubmed/?term=25544671. 10.1007/s00432-014-1900-3 PMC11823957 25544671

[22] Chan DSY, Twine CP, Lewis WG. Systematic review and meta-analysis of the influence of HER2 expression and amplification in operable oesophageal cancer. J Gastrointest Surg. 2012 Oct;16(10):1821-9. https://www.ncbi.nlm.nih.gov/pubmed/?term=22843084. 10.1007/s11605-012-1979-2 22843084

[23] Rossi E, Grisanti S, Villanacci V, Della Casa D, Cengia P, Missale G, et al. HER-2 overexpression/amplification in Barrett’s oesophagus predicts early transition from dysplasia to adenocarcinoma: a clinico-pathologic study. J Cell Mol Med. 2009;13(9B):3826–33. https://www.ncbi.nlm.nih.gov/pubmed/?term=19292734. 10.1111/j.1582-4934.2008.00517.x PMC4516530 19292734

[24] Yoon HH, Shi Q, Sukov WR, Lewis MA, Sattler CA, Wiktor AE, et al. Adverse prognostic impact of intratumor heterogeneous HER2 gene amplification in patients with esophageal adenocarcinoma. J Clin Oncol. 2012;30(32):3932–8. https://www.ncbi.nlm.nih.gov/pubmed/?term=22987085%2C. 10.1200/JCO.2012.43.1890 PMC3675687 22987085

[25] Simon R. Applications of tissue microarray technology. Methods Mol Biol. 2010;664:1-16. https://www.ncbi.nlm.nih.gov/pubmed/?term=20690047. 10.1007/978-1-60761-806-5_1 20690047

[26] Helbig D, Quaas A, Mauch C, Merkelbach-Bruse S, Büttner R, Emberger M, et al. Copy number variations in atypical fibroxanthomas and pleomorphic dermal sarcomas. Oncotarget. 2017;8(65):109457–109467. https://www.ncbi.nlm.nih.gov/pubmed/?term=29312620. 10.18632/oncotarget.22691 PMC5752533 29312620

[27] Rüschoff J, Nagelmeier I, Baretton G, Dietel M, Höfler H, Schildhaus HU, et al. Her2 testing in gastric cancer. What is different in comparison to breast cancer? Pathologe. 2010;31(3):208–217. https://www.ncbi.nlm.nih.gov/pubmed/?term=20443098. 10.1007/s00292-010-1278-1 20443098

[28] Loeser H, Waldschmidt D, Kuetting F, Heydt C, Zander T, Plum P, et al. Copy-number variation and protein expression of DOT1L in pancreatic adenocarcinoma as a potential drug target. Mol Clin Oncol. 2017;6(5):639–642. https://www.ncbi.nlm.nih.gov/pubmed/?term=28529740. 10.3892/mco.2017.1194 PMC5432215 28529740

[29] Wolff AC, Hammond MEH, Hicks DG, Dowsett M, McShane LM, Allison KH, et al. Recommendations for human epidermal growth factor receptor 2 testing in breast cancer: American Society of Clinical Oncology/College of American Pathologists Clinical Practice Guideline Update. Arch Pathol Lab Med. 2014;138(2):241–256. https://www.ncbi.nlm.nih.gov/pubmed/?term=24099077. 10.5858/arpa.2013-0953-SA PMC4086638 24099077

[30] Rajagopal I, Niveditha SR, Sahadev R, Nagappa PK, Rajendra SG. HER 2 Expression in Gastric and Gastro-esophageal Junction (GEJ) Adenocarcinomas. J Clin Diagn Res. 2015;9(3):EC06–EC10. https://www.ncbi.nlm.nih.gov/pubmed/?term=25954623. 10.7860/JCDR/2015/12581.5630 PMC4413071 25954623

[31] Hechtman JF, Polydorides AD (2012). HER2/neu gene amplification and protein overexpression in gastric and gastroesophageal junction adenocarcinoma: a review of histopathology, diagnostic testing, and clinical implications. Arch Pathol Lab Med.

[32] Birkman E-M, Ålgars A, Lintunen M, Ristamäki R, Sundström J, Carpén O (2016). EGFR gene amplification is relatively common and associates with outcome in intestinal adenocarcinoma of the stomach, gastro-oesophageal junction and distal oesophagus. BMC Cancer.

[33] Lei Y, Huang J, Zhao Q, Jiang N, Xu H, Wang Z, et al. The clinicopathological parameters and prognostic significance of HER2 expression in gastric cancer patients: a meta-analysis of literature. World J Surg Oncol. 2017;15(1):68. https://www.ncbi.nlm.nih.gov/pubmed/?term=28327158. 10.1186/s12957-017-1132-5 PMC5359900 28327158

[34] Hofmann M, Stoss O, Shi D, Büttner R, Van De Vijver M, Kim W, et al. Assessment of a HER2 scoring system for gastric cancer: Results from a validation study. Histopathology. 2008;52(7):797–805. https://www.ncbi.nlm.nih.gov/pubmed/?term=18422971. 10.1111/j.1365-2559.2008.03028.x 18422971

[35] Dulak AM, Stojanov P, Peng S, Lawrence MS, Fox C, Stewart C, et al. Exome and whole-genome sequencing of esophageal adenocarcinoma identifies recurrent driver events and mutational complexity. Nat Genet. 2013;45(5):478–86. https://www.ncbi.nlm.nih.gov/pubmed/?term=23525077. 10.1038/ng.2591 PMC3678719 23525077

[36] Cancer Genome Atlas Research Network, Analysis Working Group: Asan University, BC Cancer Agency, Brigham and Women’s Hospital, Broad Institute, Brown University, et al. Integrated genomic characterization of oesophageal carcinoma. Nature. 2017;541(7636):169–75. https://www.ncbi.nlm.nih.gov/pubmed/?term=28052061. 10.1038/nature20805 PMC5651175 28052061

[37] Kato S, Okamura R, Baumgartner JM, Patel H, Leichman L, Kelly K, et al. Analysis of circulating tumor DNA and clinical correlates in patients with esophageal, gastroesophageal junction and gastric adenocarcinoma. Clin Cancer Res. 2018 Dec 15;24(24):6248-6256. https://www.ncbi.nlm.nih.gov/pubmed/?term=30348637. 10.1158/1078-0432.CCR-18-1128 PMC6384095 30348637

[38] Lee S, de Boer WB, Fermoyle S, Platten M, Kumarasinghe MP. Human epidermal growth factor receptor 2 testing in gastric carcinoma: Issues related to heterogeneity in biopsies and resections. Histopathology. 2011;59(5):832–840. https://www.ncbi.nlm.nih.gov/pubmed/?term=22092394. 10.1111/j.1365-2559.2011.04017.x 22092394

[39] Gullo I, Grillo F, Molinaro L, Fassan M, De Silvestri A, Tinelli C, et al. Minimum biopsy set for HER2 evaluation in gastric and gastro-esophageal junction cancer. Endosc Int Open. 2015;3(2):E165–E170. https://www.ncbi.nlm.nih.gov/pubmed/?term=26135662. 10.1055/s-0034-1391359 PMC4477016 26135662

[40] Fazlollahi L, Remotti HE, Iuga A, Yang HM, Lagana SM, Sepulveda AR. HER2 Heterogeneity in gastroesophageal cancer detected by testing biopsy and resection specimens. Arch Pathol Lab Med. 2018;142(4):516–522. https://www.ncbi.nlm.nih.gov/pubmed/?term=28782986. 10.5858/arpa.2017-0039-OA 28782986

[41] Slamon DJ, Clark GM, Wong SG, Levin WJ, Ullrich A, McGuire WL. Human breast cancer: correlation of relapse and survival with amplification of the HER-2/neu oncogene. Science. 1987;235(4785):177–182. https://www.ncbi.nlm.nih.gov/pubmed/?term=3798106. 10.1126/science.3798106 3798106

[42] Ménard S, Fortis S, Castiglioni F, Agresti R, Balsari A. HER2 as a prognostic factor in breast cancer. Oncology. 2001;61 Suppl 2:67-72. https://www.ncbi.nlm.nih.gov/pubmed/?term=11694790. 10.1159/000055404 11694790

[43] Hedner C, Borg D, Nodin B, Karnevi E, Jirström K, Eberhard J. Expression and prognostic significance of human epidermal growth factor receptors 1, 2 and 3 in oesophageal and gastric adenocarcinomas preneoadjuvant and postneoadjuvant treatment. J Clin Pathol. 2018;71(5):451–462. https://www.ncbi.nlm.nih.gov/pubmed/?term=29138285. 10.1136/jclinpath-2017-204774 29138285

[44] Watson S, Validire P, Cervera P, Zorkani N, Scriva A, Lemay F, et al. Combined HER2 analysis of biopsies and surgical specimens to optimize detection of trastuzumab-eligible patients in eso-gastric adenocarcinoma: A GERCOR study. Ann Oncol. 2013;24(12):3035–3039. https://www.ncbi.nlm.nih.gov/pubmed/?term=24114855. 10.1093/annonc/mdt393 24114855

